# An Avid Imitator

**DOI:** 10.14740/jocmr2458w

**Published:** 2016-01-26

**Authors:** Dimitrios Farmakiotis, Alexis Liakos, Michael B. Miller, Jeffrey F. Krane, Lindsey R. Baden, Sarah P. Hammond

**Affiliations:** a Division of Infectious Diseases, Brigham and Women’s Hospital and Dana-Farber Cancer Institute Harvard Medical School, Boston, MA, USA; b Division of Infectious Diseases, Warren Alpert Medical School of Brown University, Providence, RI, USA; c Department of Pathology, Brigham and Women’s Hospital and Dana-Farber Cancer Institute Harvard Medical School, Boston, MA, USA

**Keywords:** *Cryptococcus*, Lymphoma, Opportunistic infections

## Abstract

We present a case of disseminated cryptococcal disease, coexisting with and mimicking lymphoma. Determination of serum cryptococcal antigen should be considered for lymphopenic patients with hematologic malignancies, presenting with unexplained fever, and/or lymphadenopathy and/or pulmonary findings. Patients with hematologic malignancies treated with chemotherapy regimens are susceptible to diverse opportunistic infections. Therefore, in this patient population, it is often necessary to obtain a definitive pathologic diagnosis, to diagnose uncommon syndromes and guide management.

## Introduction

In patients with hematologic malignancies (HMs), disease progression or relapse is frequently the most common consideration in the differential diagnosis of clinical syndromes with systemic symptoms and signs. However, patients with HM, especially those treated with intensive chemotherapy regimens, are susceptible to opportunistic bacterial, viral, but also fungal infections, including cryptococcal disease [1-3]. Herein, we present an unusual case of disseminated cryptococcosis, coexisting with and mimicking lymphoma.

## Case Report

**Figure 1:**
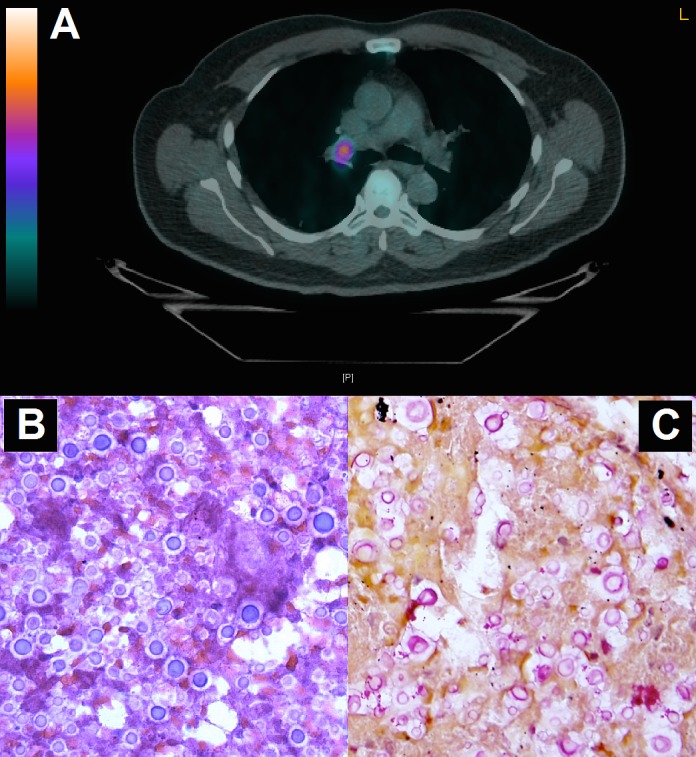
(A) PET/CT showing enlarged, FDG-avid mediastinal lymphadenopathy. (B) Hematoxylin-eosin and (C) mucicarmine stains of an endobronchial ultrasound-guided fine-needle aspirate from the mediastinal lymph node showing many encapsulated yeast forms with narrow-based budding, morphologically consistent with *Cryptococcus* species.

A 57-year-old man with non-Hodgkin’s lymphoma presented to another institution 4 days after his third cycle of rituximab, cyclophosphamide, adriamycin, vincristine and prednisone (R-CHOP) with headache, malaise and fevers up to 102 °F. A chest computerized tomography (CT) showed a right upper lobe opacity concerning for pneumonia. He received 11 days of empiric vancomycin, cefepime and doxycycline for tickborne diseases and was discharged home to complete an additional 7-day course of levofloxacin and doxycycline. He continued to feel unwell and was readmitted to our institution the following day with malaise and tachycardia. His past medical history was significant for follicular lymphoma, localized below the diaphragm with extra-nodal (lumbar spine) involvement diagnosed 4 months prior to presentation, hypertension and pulmonary embolism. He worked as a computer programmer and had not traveled outside of New England. He had no history of animal exposures. His vital signs on admission were: temperature 100.5 °F, blood pressure 130/90 mm Hg, heart rate 130/min, respiratory rate 18/min, and oxygen saturation 98% on room air; he was not in distress. He had no peripheral lymphadenopathy or skin lesions. His cardiovascular, pulmonary, abdominal and neurologic exams were unremarkable. His laboratory workup was notable for lymphopenia (absolute lymphocyte count of 250 cells/μL) and hyponatremia (125 mEq/L). Blood, urine and sputum cultures were negative. Positron emission tomography/CT (PET/CT) performed to evaluate for lymphoma progression showed enlarged, fluoro-2-deoxy-D-glucose (FDG)-avid mediastinal lymphadenopathy, which was new compared to 3 months prior, and had a maximum standardized uptake value (SUV) of 7, suggestive of active lymphoma (Fig. 1A). A bronchoscopy with ultrasound-guided fine-needle aspiration of a mediastinal lymph node was performed.

Hematoxylin-eosin (Fig. 1B) and mucicarmine (Fig. 1C) stains of an endobronchial ultrasound-guided fine-needle aspirate from the lymph node revealed many encapsulated yeast forms with narrow-based budding, morphologically consistent with *Cryptococcus* species, and no malignant cells. Serum cryptococcal antigen titer was 1:128. CD4^+^ lymphocyte count was 78 cells/μL and he was HIV negative. Although the headache that he initially presented with had resolved, a lumbar puncture was performed [1]. The opening pressure was 28 mm Hg. The cerebrospinal fluid (CSF) white blood cell count was 365 cells/μL, 87% of which were neutrophils, and protein was elevated (416 mg/dL, normal range: 40 - 70 mg/dL). Cryptococcal antigen in the CSF was also positive (1:128), and CSF cultures grew *Cryptococcus neoformans*.

Our patient received induction treatment with liposomal amphotericin-B (5 mg/kg daily) and 5-flucytosine (100 mg/kg daily) for 2 weeks [1]. His symptoms resolved, repeat CSF cultures were negative, and he transitioned to oral fluconazole 800 mg daily for 8 weeks, and 400 mg daily until completion of chemotherapy, with the plan to continue 200 mg of fluconazole daily (maintenance) indefinitely, at least until lymphocyte count recovery [1].

## Discussion

Between 1956 and 1972, the frequency of cryptococcal infection in patients with chronic lymphocytic leukemia and Hodgkin’s lymphoma was estimated to be 24.3 and 13.3 per 1,000 patients, respectively [2]. However, in a more recent cohort [3], the incidence of cryptococcosis at a large cancer center was 18/100,000 admissions, between 1989 and 1999. Furthermore, infectious lymphadenitis was observed in 3.9% of lymph node biopsies among patients with chronic lymphocytic leukemia and small-cell lymphoma at one center between 2003 and 2012, but only one case was due to *Cryptococcus neoformans* [4]. The lower rates of documented cryptococcal disease recently may be related to increased use of azole prophylaxis in cancer patients who are at risk for invasive fungal infections, particularly those with HM.

Cryptococcosis typically affects patients who have impaired cell-mediated immunity or receive high-dose steroids [3, 5-8]. In two case series of cancer patients with proven cryptococcal infection, 83% [2] and 65% [3] had lymphoproliferative disorders, most of whom had advanced cancer and were heavily treated. We know little about the risk for cryptococcosis in presumably lower net states of immunosuppression, for example with low-intensity chemotherapy, or early in the course of lymphoma treatment. In the two studies mentioned above, 100% [2] and 61% [3] of affected patients had significant lymphopenia, an important risk factor for cryptococcal disease, as exemplified in the present and previous case reports [5-7].

The clinical and radiological presentation of cryptococcosis in cancer patients is non-specific and frequently mimics progression of primary malignancy or metastatic disease [3, 5, 7, 9]. In one study of cancer patients with cryptococcal disease, pulmonary cryptococcosis was initially misdiagnosed as cancer in 35%, healthcare-associated pneumonia in 22%, and indeterminate lung nodules in 19% [3]. 18-FDG PET/CT is often used to differentiate between benign and malignant lesions, and an SUV of approximately 5 is typically considered a reliable cutoff [5]. Nevertheless, in our patient, cryptococcal involvement of lymph nodes manifested with an SUV of 7, and in another similar case with an SUV of 13 [5]. In a series of seven patients with pulmonary cryptococcal lesions, SUV values ranged between 2.2 and 11.6 [9]. It should be noted that clinical and radiological responses to chemotherapy observed in other lymphoma areas usually favor infectious causes of new FDG-avid lesions.

Immunocompromised patients may have atypical clinical and radiographic presentations of infectious diseases, so pathologic confirmation is often necessary to make a correct diagnosis. As this case demonstrates, cytologic sampling can readily demonstrate the presence of fungal organisms, without the delays involved with special stains and/or fungal cultures. Accurate diagnosis is particularly important for targeted antifungal treatment, given the emerging threat of resistance among medically important fungi, as a result of increased empiric and prophylactic use of antifungal agents [10].

In conclusion, cryptococcal disease can coexist with and mimic lymphoma. Determination of serum cryptococcal antigen should be considered for lymphopenic patients with unexplained fever, lymphadenopathy, and pulmonary findings. The present report highlights the importance of obtaining a definitive pathologic diagnosis to diagnose uncommon syndromes and guide treatment in patients with hematologic malignancies.
